# Base-Position Error Rate Analysis of Next-Generation Sequencing Applied to Circulating Tumor DNA in Non-Small Cell Lung Cancer: A Prospective Study

**DOI:** 10.1371/journal.pmed.1002199

**Published:** 2016-12-27

**Authors:** Nicolas Pécuchet, Eleonora Zonta, Audrey Didelot, Pierre Combe, Constance Thibault, Laure Gibault, Camille Lours, Yves Rozenholc, Valérie Taly, Pierre Laurent-Puig, Hélène Blons, Elizabeth Fabre

**Affiliations:** 1 INSERM UMR-S1147, CNRS SNC 5014, Equipe labélisée Ligue Contre le Cancer, Université Sorbonne Paris Cité, Paris, France; 2 Department of Medical Oncology, Hôpital Européen Georges Pompidou (HEGP), Assistance Publique—Hôpitaux de Paris, Paris, France; 3 Department of Pathology, Hôpital Européen Georges Pompidou (HEGP), Assistance Publique—Hôpitaux de Paris, Paris, France; 4 Department of Biochemistry, Pharmacogenetics and Molecular Oncology, Hôpital Européen Georges Pompidou (HEGP), Assistance Publique—Hôpitaux de Paris, Paris, France; 5 MERIT—UMR IRD 216, Université Sorbonne Paris Cité, Paris, France; MSKCC, UNITED STATES

## Abstract

**Background:**

Circulating tumor DNA (ctDNA) is an approved noninvasive biomarker to test for the presence of *EGFR* mutations at diagnosis or recurrence of lung cancer. However, studies evaluating ctDNA as a noninvasive “real-time” biomarker to provide prognostic and predictive information in treatment monitoring have given inconsistent results, mainly due to methodological differences. We have recently validated a next-generation sequencing (NGS) approach to detect ctDNA. Using this new approach, we evaluated the clinical usefulness of ctDNA monitoring in a prospective observational series of patients with non-small cell lung cancer (NSCLC).

**Methods and Findings:**

We recruited 124 patients with newly diagnosed advanced NSCLC for ctDNA monitoring. The primary objective was to analyze the prognostic value of baseline ctDNA on overall survival. ctDNA was assessed by ultra-deep targeted NGS using our dedicated variant caller algorithm. Common mutations were validated by digital PCR. Out of the 109 patients with at least one follow-up marker mutation, plasma samples were contributive at baseline (*n* = 105), at first evaluation (*n* = 85), and at tumor progression (*n* = 66). We found that the presence of ctDNA at baseline was an independent marker of poor prognosis, with a median overall survival of 13.6 versus 21.5 mo (adjusted hazard ratio [HR] 1.82, 95% CI 1.01–3.55, *p* = 0.045) and a median progression-free survival of 4.9 versus 10.4 mo (adjusted HR 2.14, 95% CI 1.30–3.67, *p* = 0.002). It was also related to the presence of bone and liver metastasis. At first evaluation (E1) after treatment initiation, residual ctDNA was an early predictor of treatment benefit as judged by best radiological response and progression-free survival. Finally, negative ctDNA at E1 was associated with overall survival independently of Response Evaluation Criteria in Solid Tumors (RECIST) (HR 3.27, 95% CI 1.66–6.40, *p* < 0.001). Study population heterogeneity, over-representation of *EGFR*-mutated patients, and heterogeneous treatment types might limit the conclusions of this study, which require future validation in independent populations.

**Conclusions:**

In this study of patients with newly diagnosed NSCLC, we found that ctDNA detection using targeted NGS was associated with poor prognosis. The heterogeneity of lung cancer molecular alterations, particularly at time of progression, impairs the ability of individual gene testing to accurately detect ctDNA in unselected patients. Further investigations are needed to evaluate the clinical impact of earlier evaluation times at 1 or 2 wk. Supporting clinical decisions, such as early treatment switching based on ctDNA positivity at first evaluation, will require dedicated interventional studies.

## Abstract 2

Hélène Blons and colleagues use next-generation sequencing to study circulating tumor DNA and implications for prognosis in non-small-cell lung cancer.

## Author Summary

**Why Was This Study Done?:**

- Plasma circulating tumor DNA (ctDNA) allows noninvasive detection of cancer mutations.
- At relapse, the acquisition of secondary mutations may guide new treatment strategies.
- Next-generation sequencing (NGS) is an emerging tool to detect ctDNA mutations.

**What Did the Researchers Do and Find?:**

- ctDNA was characterized in 109 patients with advanced non-small cell lung cancer (NSCLC) using a 22 gene-panel NGS at diagnosis, at first evaluation, and at time of progression.
- ctDNA at diagnosis was positive in 71% of patients and was related to shorter overall survival.
- Under treatment, ctDNA clearance at first evaluation was associated with objective response, longer progression-free survival, and overall survival.

**What Do These Findings Mean?:**

- ctDNA is a prognostic marker in advanced NSCLC, and its evolution reflects treatment efficacy.
- Analysis of ctDNA allows the characterization of secondary mutations at progression.
- A key limitation of monitoring ctDNA is its lack of detection at diagnosis or at time of progression in some patients; therefore, it cannot replace tumor biopsy analysis or radiological evaluation, but it contributes complementary information.

## Introduction

Lung cancer is the leading cause of cancer-related death worldwide, with 1.8 million new cases in 2012 [1]. More than 50% of lung cancer patients are diagnosed with metastatic disease and have a 5-y survival rate of <5% [2]. However, the development of novel therapeutic approaches based on predictive biomarkers and the use of targeted therapies have improved the clinical outcome of advanced non-small cell lung cancer (NSCLC) patients [3]. The list of molecular targets is increasing rapidly in adenocarcinomas (*EGFR*, *ALK*, *HER2*, *BRAF*, *MET*, *ROS1*, and *RET*) and to a lesser extent also in squamous-cell carcinomas (*FGFR1* and *PIK3CA*) [4]. Molecular testing is routinely performed on DNA extracted from tumor tissue, i.e., solid biopsy, and more recently on circulating cell-free DNA, i.e., liquid biopsy [5]. Circulating DNA may facilitate the study of spatial and temporal tumor heterogeneity [6,7], the characterization of genetic changes under treatment, and the identification of secondary resistance mechanisms [8,9]. It is also a good candidate for early evaluation of treatment efficacy because of its rapid clearance from plasma [10,11].

In lung cancer, circulating tumor DNA (ctDNA) has been validated as a surrogate material to detect mutations in the gene encoding epidermal growth factor receptor (*EGFR*) at diagnosis [12–16] and to identify secondary *EGFR* mutations, such as p.T790M or p.C797S, at relapse in patients treated with EGFR inhibitors [16–20]. The prognostic value of ctDNA has been investigated in different cancer types and is becoming an important topic in lung cancer. The technical challenge of this research is to detect low concentrations of ctDNA, as found in lung cancer, and in clinical situations such as the presence of a low tumor burden. Indeed, by analysis of mutations, ctDNA was identified in only 62% of *EGFR*-mutated lung cancer patients [12], as compared to 92% of *KRAS*-mutated colon-cancer patients [21]. To accurately detect ctDNA, ultra-sensitive methods such as digital PCR (dPCR) [22,23] or optimized ultra-deep next-generation sequencing (NGS) are required [24–26]. Two issues have to be considered: (1) plasma contains low concentrations of cell-free DNA, and (2) ctDNA is often present at a low allelic ratio. The main interest in NGS is in allowing a broad molecular screen using moderate amounts of template DNA. Such an approach is well adapted to the heterogeneous molecular mechanisms driving lung carcinogenesis, tumor progression, and acquired resistance to therapy. NGS will also allow a wide genetic analysis suitable for unselected patient screening. Moreover, in some specific cases, it can be used to assess the relative disposition of different genetic alterations, such as the *cis* or *trans* positions of *EGFR* p.C797S and p.T790M [18]. For this purpose, we previously developed the base-position error rate (BPER) method, a bioinformatics analytical pipeline dedicated to routine ctDNA testing using ultra-deep targeted NGS at 10,000× (Ion Proton, Thermo Fisher Scientific) [27].

In the present prospective observational study, we tested the clinical utility of liquid biopsy in advanced or metastatic NSCLC patients (*n* = 256 plasma samples from 124 patients). Patients’ inclusion was not based on the existence of a defined tumor mutation such as *EGFR* or *KRAS*. Our primary objective was to evaluate the prognostic value of ctDNA positivity before treatment initiation with respect to overall survival (OS). Secondary objectives were to evaluate the prognostic impact of ctDNA concentration at treatment initiation and under treatment. To this end, we quantified the absolute concentration of ctDNA at baseline, at first evaluation (E1), and at time of radiological progression. Finally, we investigated the occurrence of secondary mutations possibly related to treatment resistance.

## Materials and Methods

### Patients and Tumor Material

We conducted a prospective, single-centre observational study to evaluate the prognostic value of ctDNA in NSCLC patients. The research was conducted according to the recommendations outlined in the Declaration of Helsinki. The study and written protocol were approved by the relevant Ethics Committee (CPP Ile-de-France II n°2013-06-21 SC). The prospective study plan was respected with regards to the primary objective and all secondary objectives presented here. All patients signed a written informed consent form. Patients with newly diagnosed advanced or metastatic NSCLC and undergoing first-line treatment (*n* = 124) were recruited in the European Georges Pompidou Hospital medical oncology department between June 2013 and November 2015. In order to avoid potential selection bias, patients could be included before molecular testing. Exclusion criteria were previous cancer diagnosed within the last 5 y, inability to undergo medical follow-up, and inability to read or understand the consent form.

Blood samples were collected at baseline (T0) before initiation of therapy (chemotherapy or tyrosine-kinase inhibitor), at first evaluation (6 ± 2 wk; E1), and at time of tumor progression (ToP). Among 109 patients eligible for ctDNA follow-up, 85 underwent their first evaluation at 6 ± 2 wk, 13 received no follow-up evaluation, 7 had their first evaluation either before 1 mo or after 2 mo, and 4 died within the first month. Blood samples were processed within 2 h, and plasma was immediately stored frozen. Clinical data collected prospectively included sex, age, performance status (WHO), smoking history, tumor histological type, TNM tumor stage according to the 7th edition of the Union for International Cancer Control (UICC) classification, description and number of metastatic sites, treatment drugs, dates of initiation and end of treatment, radiological evaluation performed every 2 mo by CT-scan according to the Response Evaluation Criteria in Solid Tumors (RECIST) 1.1, date of progression, and date of death or last follow-up. Tumor burden was estimated using the RECIST baseline sum of longest diameters and categorized as low (≤7.5 cm) or high (>7.5 cm) as previously described [28].

### DNA Extraction from Tumor and Plasma Samples

The QIAmp Circulating Nucleic Acid Kit, QIAamp DNA Mini Kit for FFPE, and QIAamp DNA Blood Mini Kit were used for DNA extraction from 2 mL of plasma, formalin-fixed, paraffin-embedded (FFPE) tumor samples, and cell lines, respectively, according to the manufacturer’s instructions (QIAGEN, Les Ulis, France). DNA was quantified using a Qubit 2.0 Fluorometer with a Qubit dsDNA BR Assay Kit for DNA from cell lines and HS Assay Kit for circulating cell-free DNA (Life Technologies–Thermo Fisher Scientific, Saint Aubin, France). All DNA samples used in the study were stored at −20°C before use.

### dPCR: Emulsion Generation, Thermal Cycling, and Droplet Analyses

The RainDrop Digital PCR System (RainDance Technologies) was used for the dPCR. Wild-type genomic DNA and cell-line DNA were used as internal controls (S1 Table). The PCR mix was prepared as shown in S2 Table. Fragmented 20-ng DNA from negative and positive controls (Covaris S220 sonicator) and 3–6 μL of plasma DNA were added to the mix prior to compartmentalization into droplets using the RainDrop Source instrument. The samples were thermal cycled (S3 Table) using a BioRad thermal cycler (MJ-Mini, S1000 or C1000 touch). Droplets were loaded into the RainDrop Sense instrument and analyzed using RainDrop Analyst software. To calculate the percentage of mutations, a limit of blank was determined for each assay [29] and applied to all samples as previously described [27].

### NGS Analyses and Protocol

Sequencing libraries were prepared from tumor FFPE DNA and from circulating free DNA using Ion AmpliSeq Colon and Lung Cancer Research Panel v2 (Life Technologies–Thermo Fisher Scientific), following the manufacturer’s recommendations. The multiplex barcoded libraries were generated with the Ion AmpliSeq Library Kit v2 using 6 μL of plasma cell-free DNA as the input, corresponding to a median of 7.8 ng (range: 1.38–300 ng). Libraries were normalized using the Ion Library Equalizer Kit. The pooled barcoded libraries (maximum: 96) were processed on an Ion Chef System using an Ion PI Hi-Q Chef Kit and then sequenced on an Ion Proton System using an Ion PI Chip Kit v3. The FASTQ sequencing data were processed and aligned to the human genome (hg19) using the Ion-Torrent Suite v4.2.1. The BAM files generated by the Ion Torrent Suite were recalibrated using GATK v3.4–46 for local realignment around indels and base quality recalibration [30]. We then applied the BPER method to the recalibrated BAM files as previously described. This method detects high sensitivity mutations in circulating DNA at allele frequencies of as low as 0.001 for insertions or deletions >2 bp, and at 0.003 for single nucleotide variations. The BPER method is highly consistent with dPCR for *EGFR* and *KRAS* mutations, kappa 0.90 (0.73–1.06) [27]. It has been implemented within an R package entitled “PlasmaMutationDetector,” which is publicly available at https://cran.r-project.org/package=PlasmaMutationDetector. We recommend recalibrating the BAM files with GATK [30] before applying “PlasmaMutationDetector.”

### Absolute Quantification of ctDNA

ctDNA was quantified using the number of wild-type droplets, the mutation allele frequency (measured by dPCR or NGS), and the DNA extract volume (50 μL), and was normalized to 1 mL plasma. ctDNA concentration was categorized into tertiles defining low (<0.027 ng/mL), intermediate (0.027–0.50 ng/mL), and high (>0.50 ng/mL) concentrations.

### Proliferative Index

Paraffin-embedded tissues were cut into 4-μm sections and placed on Superfrost Plus slides. Samples were incubated for 40 min with the Ki67 antibody (mouse monoclonal antibody, MIB-1 clone from Dako) after an 8-min antigen retrieval step at 95°C in a BenchMark ULTRA IHC Staining Module, which was revealed with DAB peroxidase. The percentage of stained nuclei was assessed on ten adjacent high-power field sections, or on the whole sample for small biopsies. If the staining was heterogeneous, the most proliferative area was taken into account.

### Statistical Analysis

The sample size (*n* = 102) was calculated to allow detection of an OS difference with a hazard ratio (HR) of 2 between patients with and without ctDNA at baseline, considering a proportion of positive ctDNA patients of 0.6, an overall 2-y survival rate of 0.20, a power of 0.80, and type I error rate of 0.05. Baseline ctDNA was considered positive if detected by NGS or by dPCR. Statistical analyses were performed on the per-protocol population composed of all patients with at least one molecular alteration identified by NGS in the tumor or in the initial plasma sample (*n* = 109).

The cut-off date for analysis was May 2016. Patients were censored at last follow-up. Follow-up time was calculated using the reverse Kaplan-Meyer method. The OS was calculated from the date of treatment initiation until death from any cause. Progression-free survival was calculated from the date of treatment initiation until RECIST radiological progression or death. The Cox proportional-hazards regression model was used to perform univariate and multivariate analyses with a 95% confidence interval (CI). Multivariate analysis was performed using variables associated with the outcome in univariate analysis at a *p*-value of *<* 0.05.

All statistical analyses were performed using JMP software version 10.0 (SPSS, Chicago, Illinois). A *p*-value < 0.05 was considered significant.

## Results

**Fig 1:**
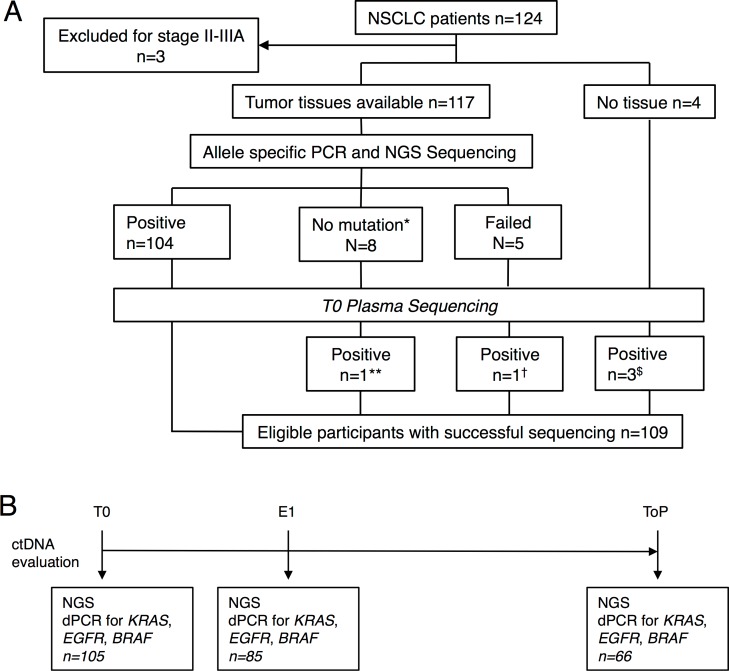
Study design. (A) Flowchart of the study population. (B) Time point of ctDNA follow-up. *Includes ALK fusion (*n* = 3) and ROS1 fusion (*n* = 1). **PIK3CA p.H1047L. ^†^TP53 p.Gly244Cys. ^$^KRAS p.G12V, TP53 p.C135Y, and TP53 p.R248W. No mutations were found for *AKT1*, *ERBB2*, *FBXW7*, *FGFR2*, *MET*, or *NOTCH1*.

The flowchart (Fig 1A) describes selection of the 109 patients analyzed in this study starting from the total population (*n* = 124). These 109 patients met all the criteria for analysis and follow-up with an identified marker mutation at baseline either in tumor tissue (*n* = 104) or in plasma at T0 (*n* = 5). It is noteworthy that *ALK* or *ROS1* rearrangements cannot be assessed by this NGS panel. As no other marker alteration was identified in patients with *ALK* and *ROS1* tumors, they were excluded. The ctDNA was evaluated at three time points: baseline (T0), first evaluation (i.e., 6 ± 2 wk, E1), and time of progression (ToP) (Fig 1B). Patients' characteristics are described in Table 1.

**Table 1:** Summary of baseline patient and tumor characteristics.

Characteristics		
	*n* / 109	%
**Sex**		
Male	49	45
Female	60	55
**Age**		
<70 y	67	61
≥70 y	42	39
**Smoking History**		
Present or former	73	67
Never	36	33
**Performance Status (WHO)**		
0–1	71	65
2–3	38	35
**Histological Types**		
Nonsquamous NSCLC	98	90
Squamous NSCLC	11	10
**Tumor Stage (UICC 7th ed.)**		
IIIB	12	11
IV	97	89
**Tumor Burden (BSLD)**		
≤7.5 cm	66	63
>7.5 cm	39	37
**Metastatic Sites**		
Bone	52	48
Liver	9	8
Brain	22	20
**Mutations**		
*TP53*	65	60
*EGFR*	47	43
*KRAS*	29	27
**Treatment Type**		
Chemotherapy	73	67
EGFR TKI	36	33

BSLD, baseline sum of longest diameters; TKI, tyrosine kinase inhibitor.

### NGS Method for ctDNA Detection

**Fig 2:**
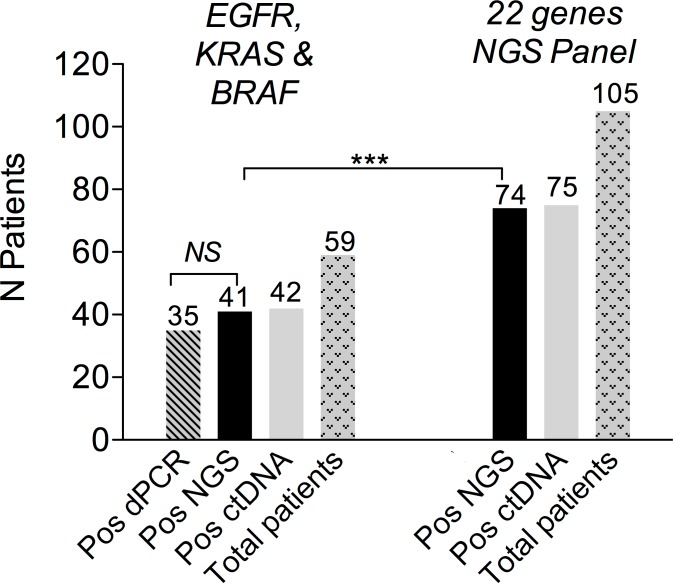
Baseline ctDNA detection using NGS. On the left, the figure shows *EGFR*, *KRAS*, and *BRAF* mutation testing with dPCR and NGS. On the right, the figure shows the 105 patients eligible for baseline ctDNA NGS testing, among whom 74 were positive.

A previous assessment of NGS performance in clinical samples showed a high level of agreement with dPCR for *EGFR* and *KRAS* mutations [27]. In the present study, double testing of 59 patients with *EGFR*-, *KRAS*-, or *BRAF*-mutated tumors confirmed these results (Fig 2). The number of patients eligible for NGS ctDNA screening was 105, as compared to 59 when considering only the three recurrent driver alterations (Fig 2). The high frequency of *TP53* mutations accounted for most of the gain in eligible patients (S1 Fig).

### Baseline ctDNA and Prognosis

**Fig 3:**
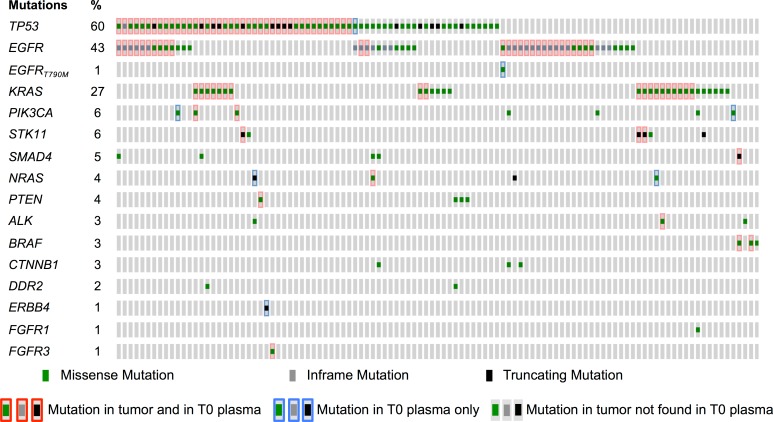
Mutations identified in tumor and in baseline plasma samples. Comparison of molecular alterations found in tumor tissue and/or in baseline (T0) plasma (*n* = 109 participants).

Of 109 patients, 4 did not have ctDNA evaluated at baseline. The plasma ctDNA detection rate was 75 (74 by NGS and 1 rescued by dPCR) out of 105 patients (71.4%, 95% CI 60%–82%, Table 2) at baseline, including 42.9%, 27.6%, and 0.9% with one, two, and three mutations, respectively. Negative baseline ctDNA was associated with a lower incidence of bone metastasis (odds ratio [OR] 0.34 [95% CI 0.14–0.83]) and lower tumor burden (OR 0.24 [95% CI 0.08–0.70]). Nine mutations (8 patients) that were not present in the tumor were detected in ctDNA at T0: one *EGFR* exon 19 deletion, one *EGFR* p.T790M mutation, two *TP53* mutations, two *PTEN* mutations, one ERBB4 mutation, and two *PIK3CA* mutations (Fig 3). Technical issues and low-quality FFPE DNA likely represent the main explanations for these discrepancies, although tumor heterogeneity cannot be ruled out.

**Table 2:** Plasma characteristics.

Treatment Time	Baseline (T0)		First Evaluation (E1)		Time of Progression (ToP)	
Characteristics	*n* plasma / 109	Median (IQR] or % (95% CI)	*n* plasma / 109	Median (IQR) or% (95% CI)	*n* plasma / 109	Median (IQR) or % (95% CI)
ctDNA positivity rate (%)	105	71.4% (60–82) (*n* = 75)	85	32% (19–45) (*n* = 27)	66	71.2% (57–83) (*n* = 47)
ctDNA concentration (ng/mL plasma)	105	0.12 (0–0.79)	85	0 (0–0.06)	46	0.09 (0.009–1.23)

IQR, interquartile range.

**Fig 4:**
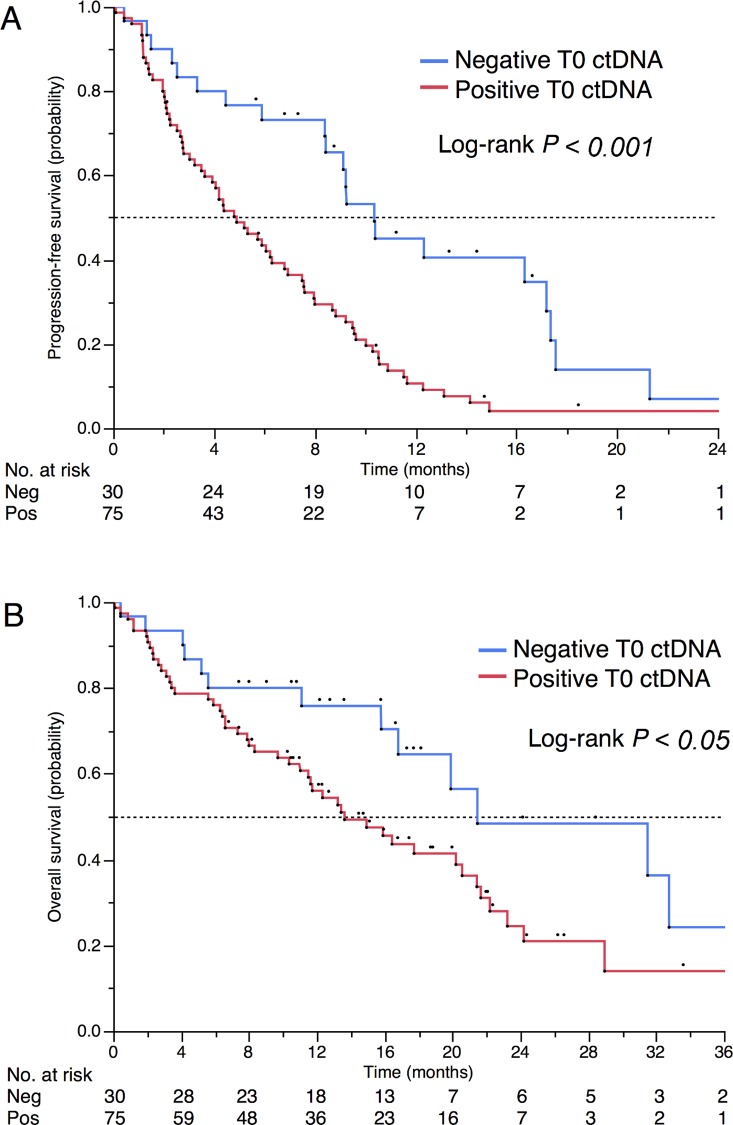
Prognostic impact of positive baseline ctDNA. (A) PFS and (B) OS in patients with positive and negative ctDNA (*n* = 105).

After a median follow-up of 18.8 mo, 94 and 63 events occurred for progression-free survival (PFS) and OS, respectively. Baseline ctDNA positivity was associated with reduced OS (median 13.6 versus 21.5 mo, *p* = 0.03, Fig 4) and poor PFS (median 4.9 versus 10.4 mo, *p* < 0.001). ctDNA remained associated with a poor outcome in multivariate analyses. HRs were 1.82 (95% CI 1.01–3.55, *p* = 0.045; Table 3) and 2.14 (95% CI, 1.30–3.67 *p* = 0.002; S4 Table) for OS and PFS, respectively. For OS, ctDNA was independent of performance status, while for PFS, ctDNA was the only prognostic factor that remained independent after adjustment on univariate significant parameters.

**Table 3:** Effect of patient and tumor baseline characteristics on OS (n = 109).

Characteristics			Univariate Cox Model			Multivariate Cox Model*		
	*n*	%	HR	95% CI	*p*	HR	95% CI	*p*
**Sex**								
Male	49	45	1			1		
Female	60	55	0.58	0.35–0.98	0.04	0.65	0.38–1.09	0.10
**Age**								
<70 y	67	61	1					
≥70 y	42	39	1.11	0.66–1.85	0.68			
**Smoking History**								
Present or former	73	67	1					
Never	36	33	0.64	0.36–1.09	0.10			
**Performance Status (WHO)**								
0–1	71	65	1			1		
2–3	38	35	2.76	1.66–4.56	0.0001	2.41	1.42–4.04	0.001
**Histological Types**								
Nonsquamous NSCLC	98	90	1					
Squamous NSCLC	11	10	1.47	0.56–3.17	0.39			
**Tumor Stage (UICC 7th ed.)**								
IIIB	12	11	1					
IV	97	89	1.19	0.52–3.43	0.70			
**Tumor Burden (BSLD)**								
≤7.5 cm	66	63	1					
>7.5 cm	39	37	1.38	0.82–2.29	0.22			
**Metastatic Sites**								
Bone	52	48	1.07*	0.65–1.76	0.79			
Liver	9	8	2.35*	1.02–4.69	0.04	2.07*	0.90–4.21	0.08
Brain	22	20	1.62*	0.86–2.88	0.13			
**Mutations**								
*TP53*	65	60	1.49*	0.89–2.57	0.13			
*EGFR*	47	43	0.64*	0.37–1.07	0.09			
*KRAS*	29	27	1.37*	0.75–2.37	0.29			
**Baseline ctDNA**								
Negative	30	29	1			1		
Positive	75	71	1.97	1.09–3.81	0.02	1.82	1.01–3.55	.045

*Relative to patients without the specific characteristic. BSLD, baseline sum of longest diameters.

**Fig 5:**
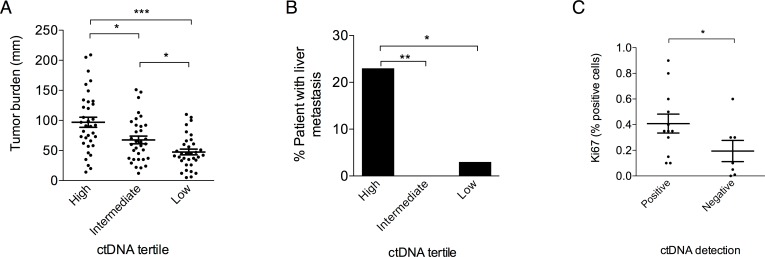
Clinical characteristics associated with ctDNA concentration. Correlation between T0 ctDNA concentration tertiles and (A) tumor burden defined by the sum of the RECIST target lesions (Mann-Whitney test) and (B) presence of liver metastasis (Fisher’s exact test). (C) Correlation between positive ctDNA at baseline and Ki67 proliferative index expressed as a % of positive cells in a subset of tumors with available tissue (*n* = 19, Mann-Whitney test). *p < 0.05, **p < 0.005, ***p < 0.001.

To study the impact of ctDNA concentration, patients were categorized into tertiles (*n* = 35 patients in each tertile) defining low (<0.027 ng/mL), intermediate (0.027–0.50 ng/mL), and high (>0.50 ng/mL) concentration groups. High ctDNA concentration was associated with higher tumor burden as evaluated by RECIST criteria (Fig 5A) and with the presence of liver metastases (Fig 5B). A multivariate analysis using an ordinal logistic model showed that tumor burden and liver metastasis were independently associated with ctDNA concentration (*p* < 0.001 and *p* < 0.008, respectively). Moreover, ctDNA positivity was associated with a higher proliferative index (Fig 5C). Concerning prognosis, the median OS was 13.0, 13.4, and 21.5 mo (*p =* 0.03), and the median PFS was 4.1, 5.7 and 10.4 mo (*p* < 0.001) for the high, intermediate, and low groups, respectively (S2 Fig).

### Monitoring Early Tumor Response Using ctDNA

**Fig 6:**
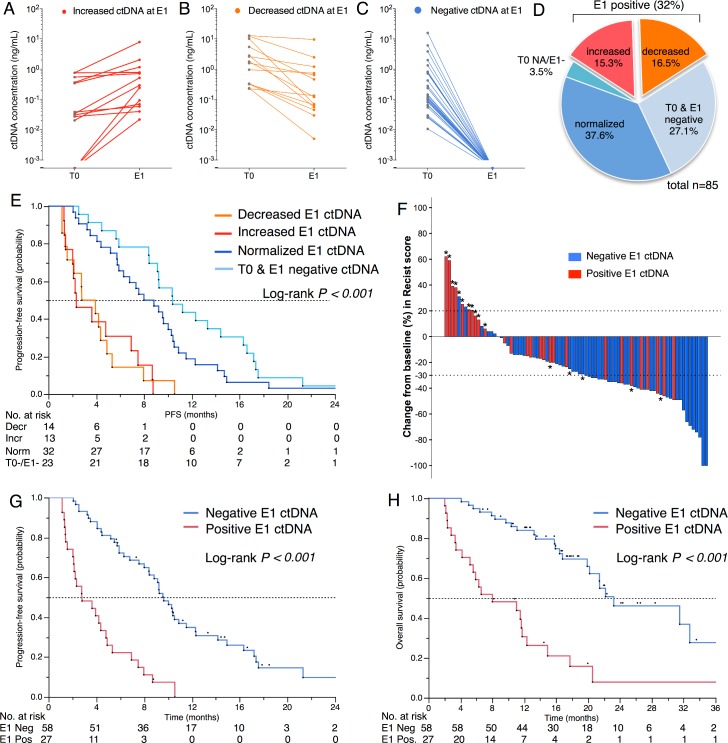
Measurement of ctDNA at first evaluation. Evolution of ctDNA concentration (ng/mL) between baseline (T0) and first evaluation (E1) showing a (A) decrease, (B) increase, (C) normalization, or (D) negativity at both time points. (E) PFS according to ctDNA evolution between T0 and E1. (F) Waterfall plot for the best changes in RECIST scores (%) according to the E1 ctDNA status. *Indicates a progressive disease as best response. (G) PFS for E1 ctDNA groups. (H) OS for E1 ctDNA groups.

The number of patients with positive ctDNA at first evaluation (E1) was 27/85 (31%), which is lower than at T0 (Table 2). Among the positive samples, ctDNA concentration had increased in 13 patients (Fig 6A) and decreased in 14 (Fig 6B). Among the negative samples (*n* = 58), the ctDNA concentration had normalized in 32 patients (Fig 6C) and remained negative in 23 patients (Fig 6D). Fig 6E shows that the negativity of ctDNA at E1 and not its decrease was the best prognostic marker for PFS. As expected, E1 ctDNA positivity was associated with RECIST tumor progression (Fig 6F) and a shorter PFS (median 2.8 versus 9.6 mo, *p* < 0.001, Fig 6G) that translated into shorter OS (median 8.0 versus 23.2 mo, *p* < 0.001; Fig 6H). The survival impact of E1 positivity remained significant both in the subgroup of patients with *KRAS*, *EGFR*, or *TP53* mutations and in the group of patients with more than one alteration (S3 Fig). In an exploratory multivariate analysis on OS, E1 ctDNA positivity (HR 3.27, 95% CI 1.66–6.40, *p* < 0.001) was independent of the result of the first RECIST evaluation (progressive disease versus stable disease: HR 2.37, 95% CI 1.05–5.28, *p =* 0.03; progressive disease versus complete or partial response: HR 2.69, 95% CI 1.18–6.14, *p* = 0.02).

### Tumor Heterogeneity at Time of Tumor Progression (ToP)

**Fig 7:**
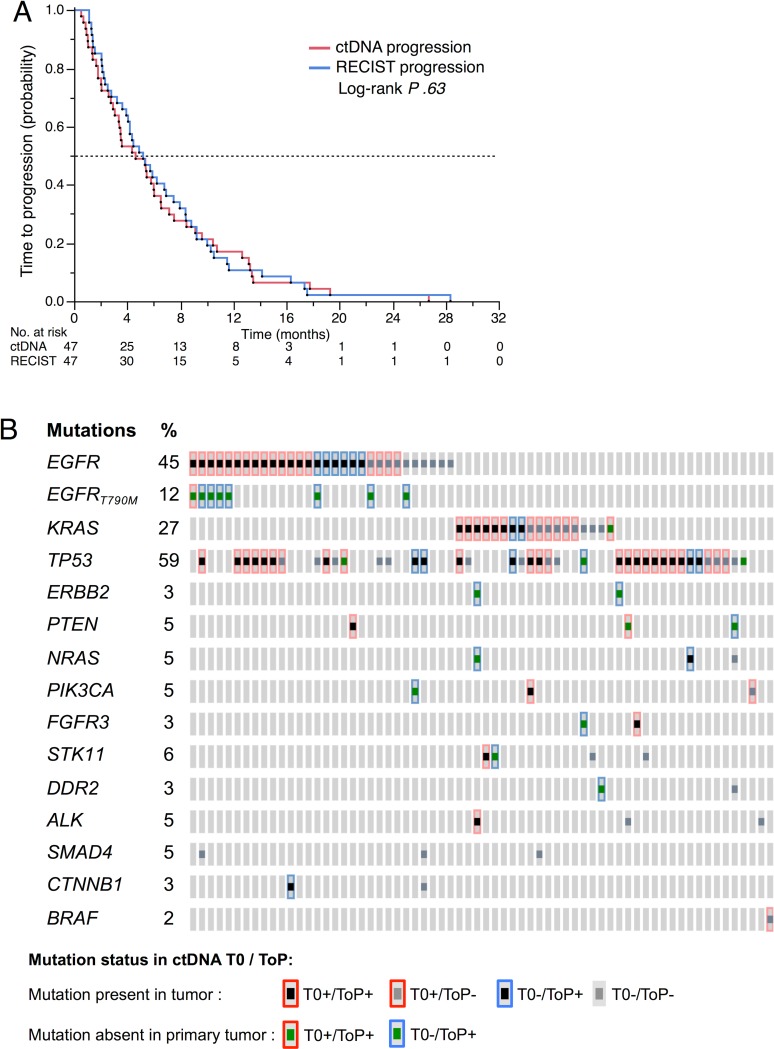
ctDNA at tumor progression. (A) Time to RECIST progression and time to ctDNA progression in 47 patients with positive ctDNA at time of progression (ToP). (B) Mutations detected in plasma at baseline (T0) and at time of progression (ToP) (*n* = 66 patients). Acquired mutations detected neither in the tumor tissue nor in the T0 plasma are represented as a green square surrounded by blue. Mutations lost at ToP are represented as a grey square.

At the ToP, ctDNA was detected in 47/66 patients (71.2%) (Table 2). The ctDNA evolution at T0, E1, and ToP is shown in S4 Fig. The mean time between biological and radiological progression was 11 ± 9.4 d (*P* .26) in a pairwise analysis of ctDNA and RECIST time to progression. The Kaplan-Meier estimation of median time to ctDNA progression was 139 d (95% CI 92–181) and the median time to RECIST progression was 156 d (95% CI 118–208) (Fig 7A).

The mutations found at progression are shown in Fig 7B, most of which were present in tumor DNA at the time of diagnosis. However, for acquired mutations, we noticed a recurrent appearance of the p.T790M mutation in the *EGFR*-mutated group but much more diverse types of mutations in the *EGFR* wild-type tumors.

## Discussion

Our study suggests that ctDNA detection using high-throughput sequencing technologies is a valuable tool to determine patient prognosis in advanced NSCLC. We have confirmed and extended the prognostic value of ctDNA previously reported by Karachaliou and colleagues in the *EGFR*-mutated subgroup population [14]. Our study shows an additive value of ctDNA in evaluating treatment efficacy when used in parallel with radiological evaluation. Moreover, we found a strong association between ctDNA at first evaluation and treatment benefit in terms of radiological response, PFS, and OS.

Our study has several limitations. Firstly, while the patients were not selected on the basis of tumor mutation, the study population is biased towards an excess of *EGFR* mutations for a white population [31]. However, one-half of the patients had non-*EGFR* mutations, and we found no difference in ctDNA detection rates between *EGFR* and non-*EGFR* mutations. The heterogeneity of our study population in regard to treatment and clinical features does limit the statistical power of our results. From a technical point of view, the NGS panel used was restricted to 22 genes. The use of a comprehensive cancer panel including genes with recurrent mutations in lung cancer, such as *KEAP1* and *NF1*, would certainly be more efficient to identify patients for whom ctDNA follow-up would be possible. Moreover, both these genes have been implicated in secondary resistance to chemotherapy and targeted therapy [32,33]. Increasing the number of markers per individual is expected to increase clinical sensitivity and specificity, as shown by Newman et al. [34]. Our bioinformatics analytical pipeline is applicable to any panel and any sequencing technology following its simple validation using a set of controls to calculate the panel’s background noise. However, gene fusions, copy number changes, and <3 base-pair indels will remain undetected.

One of the main findings of our study is that measurement of ctDNA in plasma under treatment should be interpreted per se and not relative to its baseline concentration. The absence of ctDNA normalization at the first evaluation has a major prognostic impact on both PFS and OS. The ctDNA concentration reported in our study was an absolute measurement as opposed to being a relative percentage measure as with mutant allele fraction (MAF). In some clinical conditions such as infection [35], the MAF might be biased by the presence of large quantities of nontumor circulating DNA. The present study was designed to detect small changes in ctDNA concentration in order to evaluate treatment response. We chose to report the DNA concentrations to avoid biases due to the presence of non-tumor DNA. Because ctDNA clearance appears important for clinical evaluation, MAF is also appropriate to analyze ctDNA dynamics under treatment. Our results are consistent with previous findings showing that ctDNA normalization before the third cycle of treatment in *EGFR*-mutated patients is associated with improved OS [36,37].

Further clinical studies are required to define the best time for ctDNA evaluation and to determine whether patients with positive ctDNA under one treatment might benefit from a treatment change. Previous series have demonstrated a detectable decrease in ctDNA at 15 d after treatment initiation.

It is worth noting that patients with undetectable ctDNA at baseline and those with undetectable ctDNA at first evaluation had a similar prognosis. Those showing ctDNA positivity at diagnosis could have tumors with higher proliferation capacities, as suggested by the higher Ki67 index, higher tumor burden, and increased incidence of metastasis. Concerning liver metastasis, the absence of capillary basal membrane in the liver may also facilitate the release of tumor DNA in the circulation.

In this study, the use of optimized NGS doubled the number of eligible patients for ctDNA follow-up when compared to the use of methods targeting hotspot mutations. The sensitivity of the method we developed was similar to that of dPCR in the range of DNA inputs used in the study. Our evaluations of ctDNA in a clinical setting imposed much lower DNA inputs for dPCR and NGS than is usually used in a research context. The median of 7.8 ng corresponding to 2,363 genomes that we obtained as input was insufficient to achieve a limit of detection <0.001 in all cases, yet corresponds to inputs from clinical specimens [29]. The development of automated DNA extraction methods from larger plasma volumes followed by a DNA concentration step would likely enhance ctDNA detection. The detection of ctDNA in 71% of patients was nevertheless consistent with previous studies [12]. Patients with negative or positive ctDNA at baseline had similar concentrations of total circulating DNA, thus rendering unlikely the possibility of a technical issue.

At the ToP, ctDNA was detected within the same proportion of patients as compared to baseline. The tumor heterogeneity captured by ctDNA NGS analysis was stronger for *EGFR* wild-type tumors and may reflect tobacco or chemotherapy-induced molecular heterogeneity. The sole use of dPCR limits the study to a few alterations and might not identify molecular heterogeneity at progression.

### Conclusion

This prospective study showed that ctDNA is a marker of prognosis at baseline and its normalization at first evaluation is associated with treatment benefit in metastatic NSCLC patients. Our BPER-method targeted NGS has thus been validated in a clinical setting to detect ctDNA and has allowed us to analyze ctDNA beyond *EGFR*. We believe that the added value of NGS as compared to other methods is its possible use when no tumor tissue is available. Prospective interventional studies testing the clinical impact of an early therapeutic switch based on ctDNA quantification at first evaluation are needed to fully validate our findings.

## Supporting Information

S1 Fig Detection of gene alterations in the plasma (grey) as compared to the tumor tissue (blue) at different time points: (A) baseline (T0), (B) first evaluation (E1), and (C) time of progression (ToP).
(TIF)

S2 Fig Prognostic value of ctDNA concentration. (A) PFS and (B) OS according to tertiles of baseline ctDNA concentration.
(TIF)

S3 Fig Prognostic of ctDNA positivity at first evaluation (E1). OS according to E1 ctDNA status in the subgroup of (A) KRAS-mutated patients, (B) TP53-mutated patients, (C) EGFR-mutated patients, and (D) patients with more than one mutated gene.
(TIF)

S4 Fig ctDNA evolution in 59 patients with evaluable ctDNA at T0, E1, and ToP. At the time points, 4 patients had negative ctDNA. Time of tumor progression occurred at E1 for 11 patients, which is indicated by a cross.
(TIF)

S1 Table DNA used as internal controls for dPCR. (DOCX)

S2 Table dPCR reagent components and assay mix details. (DOCX)

S3 Table dPCR thermocycling conditions. (DOCX)

S4 Table Effect of patient and tumor baseline characteristics on PFS (*n* = 109). (DOCX)

S1 Text Translated study protocol. (DOCX)

S2 Text Strengthening the Reporting of Observational Studies in Epidemiology (STROBE) checklist. (DOC)

## Abbreviations

BPER: base-position error rate
ctDNA: circulating tumor DNA
dPCR: digital PCR
E1: first evaluation
FFPE: formalin-fixed, paraffin-embedded
HR: hazard ratio
MAF: mutant allele fraction
NGS: next-generation sequencing
NSCLC: non-small cell lung cancer
OR: odds ratio
OS: overall surviva
PFS: progression-free survival
RECIST: Response Evaluation Criteria in Solid Tumors
STROBE: Strengthening the Reporting of Observational Studies in Epidemiology
T0: baseline
ToP: time of tumor progression
UICC: Union for International Cancer Control
