# Supplementary figures and images for: Base-Position Error Rate Analysis of Next-Generation Sequencing Applied to Circulating Tumor DNA in Non-Small Cell Lung Cancer: A Prospective Study
Source: PLoS Med. 2016 Dec 27;13(12):e1002199. doi: 10.1371/journal.pmed.1002199 (PMC5189949; doi:10.1371/journal.pmed.1002199)

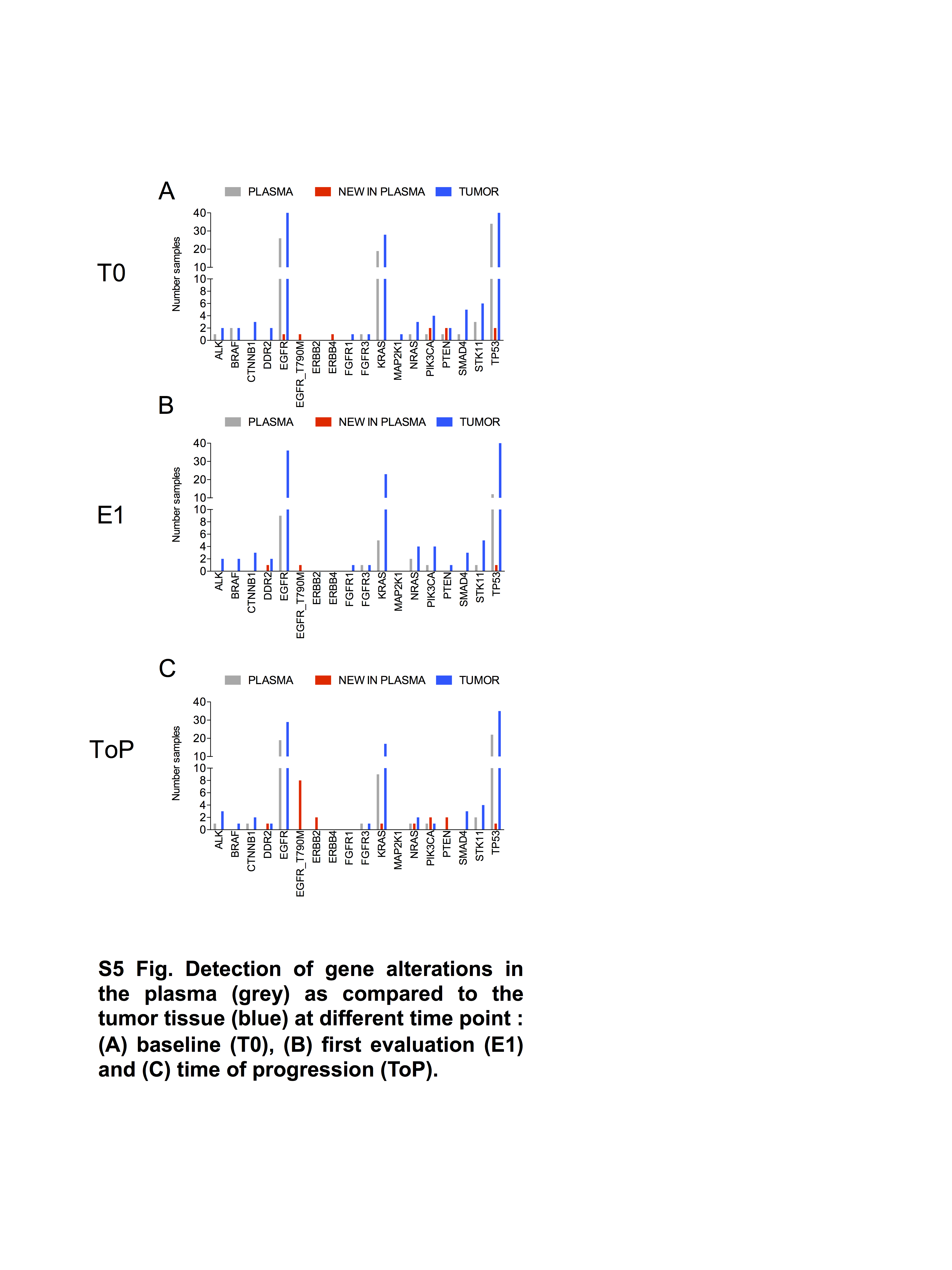

Supplement: S1 Fig — Detection of gene alterations in the plasma (grey) as compared to the tumor tissue (blue) at different time points: (A) baseline (T0), (B) first evaluation (E1), and (C) time of progression (ToP). (TIF) [file pmed.1002199.s001.tif]

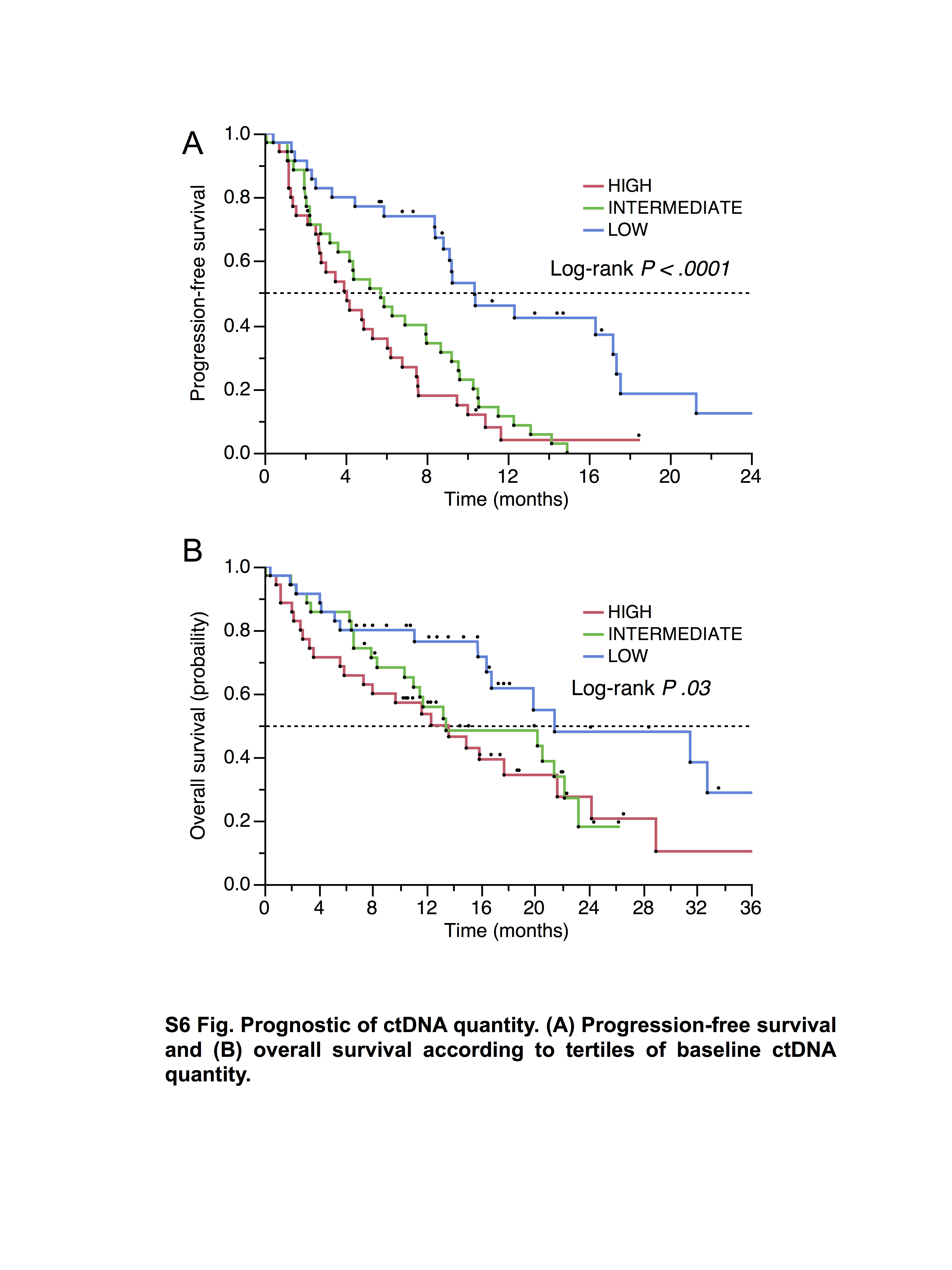

Supplement: S2 Fig — (A) PFS and (B) OS according to tertiles of baseline ctDNA concentration. (TIF) [file pmed.1002199.s002.tif]

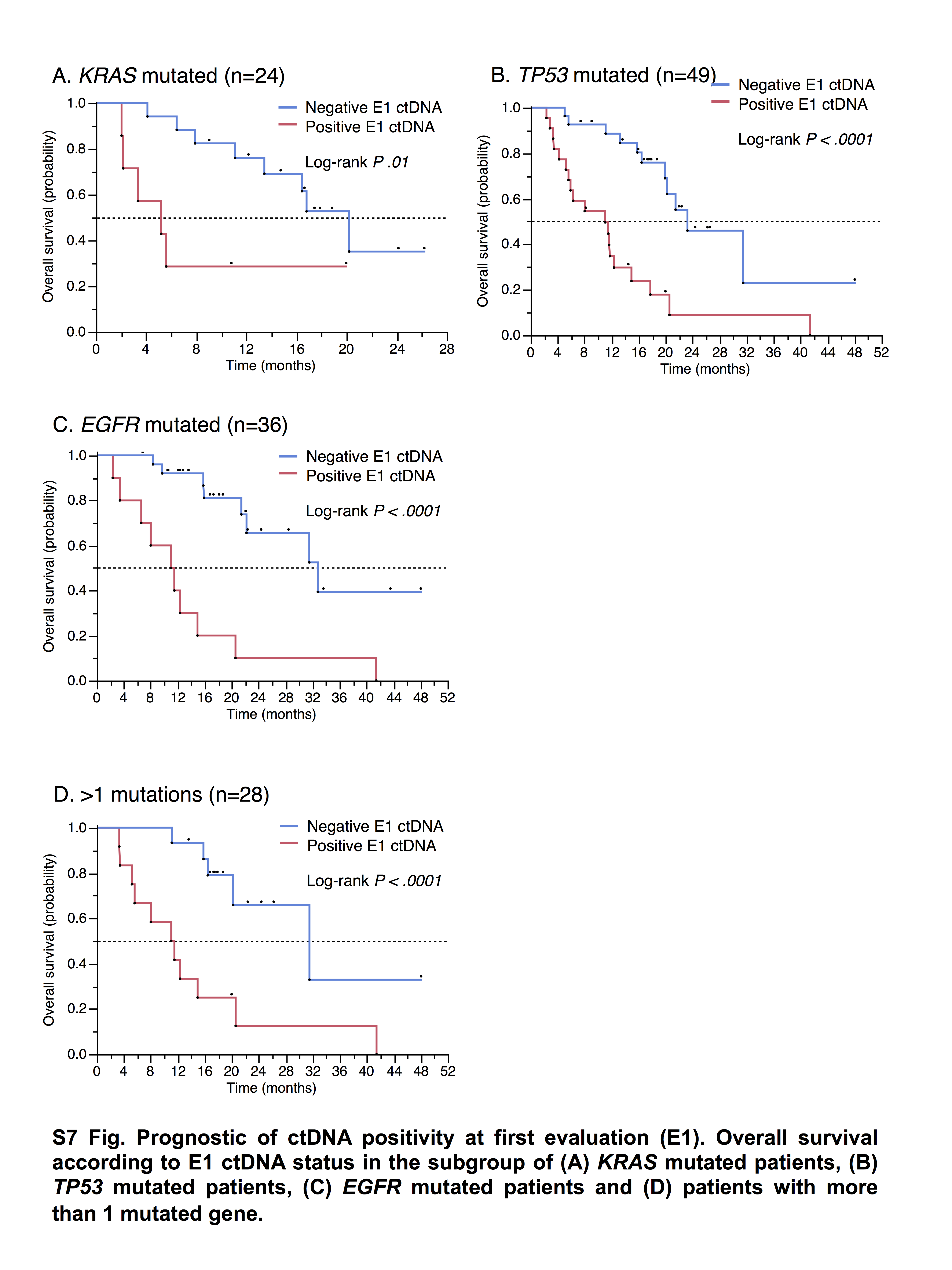

Supplement: S3 Fig — OS according to E1 ctDNA status in the subgroup of (A) KRAS-mutated patients, (B) TP53-mutated patients, (C) EGFR-mutated patients, and (D) patients with more than one mutated gene. (TIF) [file pmed.1002199.s003.tif]

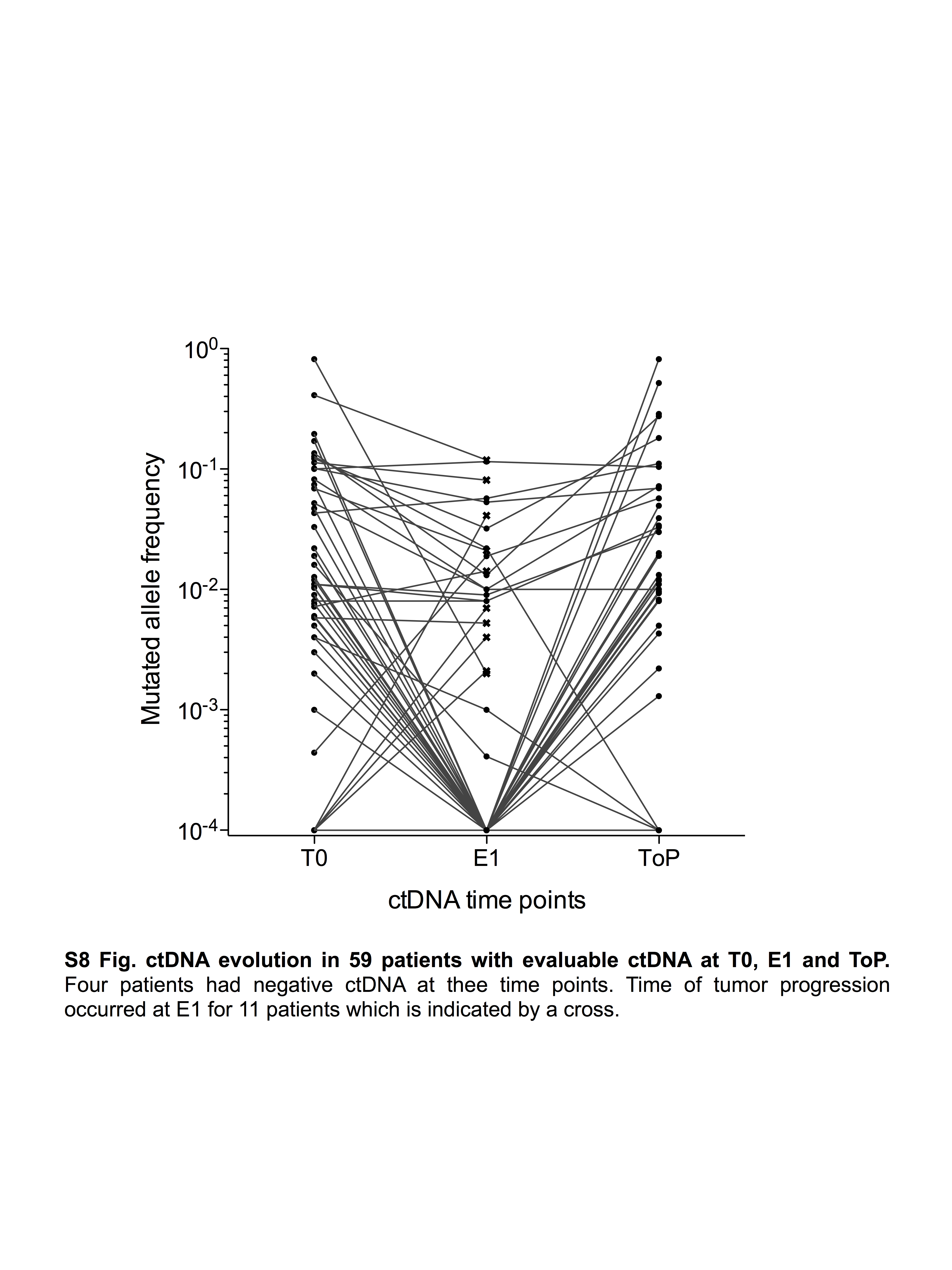

Supplement: S4 Fig — At the time points, 4 patients had negative ctDNA. Time of tumor progression occurred at E1 for 11 patients, which is indicated by a cross. (TIF) [file pmed.1002199.s004.tif]
