# Supplementary material for: Base-Position Error Rate Analysis of Next-Generation Sequencing Applied to Circulating Tumor DNA in Non-Small Cell Lung Cancer: A Prospective Study
Source: PLoS Med. 2016 Dec 27;13(12):e1002199. doi: 10.1371/journal.pmed.1002199 (PMC5189949; doi:10.1371/journal.pmed.1002199)
Supplement: S1 Table — (DOCX) [file pmed.1002199.s005.docx]

| **DNA origin** | **Mutation(s)** | **Vendors and catalogue numbers** |
| --- | --- | --- |
| genomic DNA | - | Promega (G3041) |
| H1975 | EGFR: p.L858R, p.T790M | ATCC (CRL-5908) |
| H1650 | EGFR: Del19 | ATCC (CRL-5883) |
| A427 | KRAS: p.G12D | ATCC (HTB-53) |
| patient's DNA | KRAS: p.G12R | - |
| H358 | KRAS: p.G12C | ATCC (CRL-5807) |
| SW620 | KRAS: p.G12V | ATCC (CCL-227) |
|  |  |  |
| **S1 Table. DNA used as internal controls for digital PCR** | | |
