# Supplementary material for: Base-Position Error Rate Analysis of Next-Generation Sequencing Applied to Circulating Tumor DNA in Non-Small Cell Lung Cancer: A Prospective Study
Source: PLoS Med. 2016 Dec 27;13(12):e1002199. doi: 10.1371/journal.pmed.1002199 (PMC5189949; doi:10.1371/journal.pmed.1002199)
Supplement: S2 Table — (DOCX) [file pmed.1002199.s006.docx]

| **Reagents** | **Final concentration** | **Vendors and catalogue numbers** |
| --- | --- | --- |
| TaqMan® Genotyping Master Mix | 1X | Life Technologies (4371355) |
| dNTPs | 1.2 mM | Life Technologies (R1121) |
| MgCl2 | 0.5 mM | Life Technologies (R0971) |
| Droplet Stabilizer | 1X | RainDance Technologies (20-00803) |
| TaqMan® probes | 0.2 μM | Life Technologies |
| Olinucleotide primers | 0.8 μM | Life Technologies |
| castPCR™probes | 1X VIC assay, 1X FAM assay | Life Technologies |
| ZEN probes | 0.2 μM | IDT |
| Olinucleotide primers | 0.4 μM | IDT |
| Tris-HCl pH8 10 mM | to volume | Life Technologies (AM9855G) |
| DNA (WT or mutant) | ≈ 300 to 800 copies/μL | Promega, ATCC or patient's DNA |
|  |  |  |
| **Assay name** | **Assay type** | **Vendors and catalogue numbers** |
| EGFR Ref | castPCR™ probes | Life Technologies (4465807) |
| EGFR Del19 | castPCR™ probes | Life Technologies (4465805) |
| EGFR p.L858R | castPCR™ probes | Life Technologies (4465804) |
| EGFR p. L861Q | castPCR™ probes | Life Technologies (4465804) |
| EGFR p.T790M | ZEN™ probe | IDT (custom) |
| KRAS p.G12D | TaqMan® probes | Life Technologies (custom) |
| KRAS p.G12R | TaqMan® probes | Life Technologies (custom) |
| KRAS p.G12C | TaqMan® probes | Life Technologies (custom) |
| KRAS p.G12V | TaqMan® probes | Life Technologies (custom) |
|  |  |  |
| **S2 Table. Digital PCR reagent components and assay mix details** | | |
| Reagents were added to a final volume of 25 μL. | |  |
