# Supplementary material for: Base-Position Error Rate Analysis of Next-Generation Sequencing Applied to Circulating Tumor DNA in Non-Small Cell Lung Cancer: A Prospective Study
Source: PLoS Med. 2016 Dec 27;13(12):e1002199. doi: 10.1371/journal.pmed.1002199 (PMC5189949; doi:10.1371/journal.pmed.1002199)
Supplement: S3 Table — (DOCX) [file pmed.1002199.s007.docx]

|  | **castPCR™ probes** | | | | **TaqMan® probes** | | | | **ZEN™ probes** | | | |
| --- | --- | --- | --- | --- | --- | --- | --- | --- | --- | --- | --- | --- |
|  | **Temp (C°)** | **Hold** | **Rampe Rate (°C/s)** | **Cycles** | **Temp (C°)** | **Hold** | **Rampe Rate (°C/s)** | **Cycles** | **Temp (C°)** | **Hold** | **Rampe Rate (°C/s)** | **Cycles** |
| Pre-heating | - | - | - | - | 50 | 2 min | 0.6 | 1 | - | - | - | - |
| Polymerase activation | 95 | 10 min | 0.6 | 1 | 95 | 10 min | 0.6 | 1 | 95 | 10 min | 0.6 | 1 |
| Denaturation Annealing & Extension | 92 | 15 s | 0.6 | 5 | 95 | 15 s | 0.6 | 45 | 95 | 15 s | 0.6 | 45 |
|  | 58 | 1 min | 0.6 |  | 64 | 1 min | 0.6 |  | 58 | 15 s | 0.6 |  |
| Denaturation Annealing & Extension | 92 | 15 s | 0.6 | 40 | - | - | - | - | 60 | 45 s | 0.6 |  |
|  | 60 | 1 min | 0.6 |  | - | - | - | - | - | - | - | - |
| Incubation | 98 | 10 min | 0.6 | 1 | 98 | 10 min | 0.6 | 1 | 98 | 10 min | 0.6 | 1 |
| Cooling | 10 | 10 min | - | 1 | 10 | 10 min | - | 1 | 10 | 10 min | - | 1 |
|  |  |  |  |  |  |  |  |  |  |  |  |  |
|  |  |  |  |  |  |  |  |  |  |  |  |  |

**S3 Table. Digital PCR thermocycling conditions.**
