# Supplementary material for: Base-Position Error Rate Analysis of Next-Generation Sequencing Applied to Circulating Tumor DNA in Non-Small Cell Lung Cancer: A Prospective Study
Source: PLoS Med. 2016 Dec 27;13(12):e1002199. doi: 10.1371/journal.pmed.1002199 (PMC5189949; doi:10.1371/journal.pmed.1002199)
Supplement: S4 Table — (DOCX) [file pmed.1002199.s008.docx]

| **S4 Table.** Effect of patients and tumors baseline characteristics on progression-free survival (n = 109). | | | | | | | | | | | | | |
| --- | --- | --- | --- | --- | --- | --- | --- | --- | --- | --- | --- | --- | --- |
| **Characteristics** |  | |  | **Progression-free**  **Univariate Cox model** | | |  | **Survival**  **Multivariate Cox model*** | | | | | |
|  | **N** | | **%** | **HR** | **95%CI** | ***P*** | |  | **HR** | | **95%CI** | | ***P*** |
| **Gender** |  | |  |  |  |  | |  |  | |  | |  |
| Male | *49* | | 45 | 1 |  |  | |  |  | |  | |  |
| Female | *60* | | 55 | 0.92 | 0.61-1.40 | *.69* | |  |  | |  | |  |
| **Age** |  | |  |  |  |  | |  |  | |  | |  |
| < 70 years | *67* | | 61 | 1 |  |  | |  |  | |  | |  |
| ≥ 70 years | *42* | | 39 | 0.91 | 0.59 -1.38 | *.66* | |  |  | |  | |  |
| **Smoking** **history** |  | |  |  |  |  | |  |  | |  | |  |
| Present or former | *73* | | 67 | 1 |  |  | |  | 1 | |  | |  |
| Never | *36* | | 33 | 0.65 | 0.41-0.99 | *.048* | |  | 0.89 | | 0.52-1.51 | | *.68* |
| **Performance status (WHO)** |  | |  |  |  |  | |  |  | |  | |  |
| 0-1 | *70* | | 65 | 1 |  |  | |  |  | |  | |  |
| 2-3 | *38* | | 35 | 1.49 | 0.96-2.27 | *.07* | |  |  | |  | |  |
| **Histological types** | | |  |  |  |  |  |  | |  | |  | |
| Non-squamous NSCLC | | *98* | 90 | 1 |  |  | |  | 1 | |  | |  |
| Squamous carcinoma | | *11* | 10 | 2.73 | 1.36-4.97 | *.007* | |  | 1.85 | | 0.75-3.67 | | *.11* |
| **Tumor stage (UICC 7^th^)** | |  |  |  |  |  | |  |  | |  | |  |
| IIIB | | *12* | 11 | 1 |  |  | |  |  | |  | |  |
| IV | | *97* | 89 | 1.11 | 0.54-2.03 | *.77* | |  |  | |  | |  |
| **Metastatic sites** | |  |  |  |  |  | |  |  | |  | |  |
| Bone | | *52* | 48 | 1.30* | 0.86-1.97 | *.21* | |  |  | |  | |  |
| Liver | | *9* | 8 | 2.22* | 1.02-4.25 | *.04* | |  | 1.71* | | 0.77-3.43 | | *.18* |
| Brain | | *22* | 20 | 1.06* | 0.61-1.73 | *.83* | |  |  | |  | |  |
| **Mutations** | | | | |  |  | |  |  | |  | |  |
| *TP53* | *65* | | 60 | 1.68* | 1.09-2.61 | *.02* | |  | 1.38* | | 0.87-2.22 | | *.17* |
| *EGFR* | *47* | | 43 | 0.62* | 0.40-0.94 | *.03* | |  | 0.78* | | 0.46-1.30 | | *.34* |
| *KRAS* | *29* | | 27 | 1.19* | 0.73-1.89 | *.48* | |  |  | |  | |  |
| **Baseline ctDNA** |  | |  |  |  |  | |  |  | |  | |  |
| Negative | *30* | | 29 | 1 |  |  | |  |  | |  | |  |
| Positive | *70* | | 71 | 2.43 | 1.50-4.10 | *.0002* | |  | 2.14 | | 1.30-3.67 | | *.002* |
| NA, not available; *relative to patients without the specific characteristic. | | | | | | | | | | | | | |
