# Supplementary material for: Base-Position Error Rate Analysis of Next-Generation Sequencing Applied to Circulating Tumor DNA in Non-Small Cell Lung Cancer: A Prospective Study
Source: PLoS Med. 2016 Dec 27;13(12):e1002199. doi: 10.1371/journal.pmed.1002199 (PMC5189949; doi:10.1371/journal.pmed.1002199)
Supplement: S1 Text — (DOCX) [file pmed.1002199.s009.docx]

**Evaluation of the prognostic value of circulating tumor DNA in advanced non-small cell lung cancer.** **A single-center prospective study.**

**RCB Registration ID Number:** **2013-A00681-44**

**Name and quality of the people coordinating the research project:**

Dr. Elizabeth Fabre and Dr. Nicolas Pécuchet (Medical Oncology Unit and INSERM U775)

Dr Hélène Blons and Professor Pierre Laurent-Puig (Biochemistry UF Pharmacogenetics and Molecular Oncology Unit and INSERM U775)

**Search Places**

Medical Oncology Service of the European Hospital Georges Pompidou, 20 rue Leblanc, 75015 Paris

UF Biochemistry Department of Molecular Oncology Pharmacogenetics and the European Hospital Georges Pompidou, 20 rue Leblanc, 75015 Paris

INSERM U775, University Center of the Holy Fathers, 45 rue des Saints Pères, 75006 Paris

**Scientific background**

The high incidence and mortality of lung cancer turns this disease to be a major public health concern. With over 30,000 new cases per year in France, according to the 2010 report of the Institute of Health Surveillance, lung cancer is the leading cause of cancer death in men after prostate cancer and the third in women after breast cancer and colorectal cancer (French National Cancer Institute, 2010) (Guerin *et al.,* 2010). On the pathological level, two main entities within the lung cancer are individualized: non-small cell lung cancer (NSCLC) accounting for 85% and small cell lung cancer (SCLC). The prognosis of patients with NSCLC is poor with 5-years survival rate of 15%. Mortality is mainly due to metastatic relapse.

In the early 2000s, the standard treatment included chemotherapy with cisplatin for patients whose condition was preserved, but its effectiveness remains modest (Goldstraw et *al.,* 2011). However, the discovery of highly specific genetic alterations in lung tumors, such as activating mutations in the EGF receptor *(EGFR)* has profoundly changed the treatment of patients with NSCLC in advanced stage. These genetic alterations guide targeted anti-tumor treatment, which was a major step in improving the care of patients with advanced stage NSCLC from 2008. The authorization to market EGFR TKI (Iressa and Tarceva) conditional on the presence of a mutation of the *EGFR* for their first line use in metastatic patients and more recently that of crizotinib for patients with tumors with ALK rearrangement are the first examples. Both ITK-EGFR are specific reversible EGFR inhibitors. By binding to the catalytic site of competitively to the ATP, they block the initiation of the cascade of protein phosphorylation and resulting inhibition of survival and cell proliferation (Lynch *et al.,* 2004 ; Shepherd *et al .,* 2005). The mechanism is identical to crizotinib is a TKI inhibitor of ALK (Kwak *et al.,* 2010).

The use of targeted therapies requires a prior screening of somatic alterations in the tumor. In France, molecular laboratories are labeled by the National Cancer Institute to perform these molecular testing in a clinical routine for NSCLC patients. These alterations are specific to the tumor tissue they are a tool of choice as predictive biomarkers (aid treatment choice) or prognostic (signing the severity of the disease) useful in the therapeutic management of patients. Almost all of initially responding patients treated with targeted therapy (ei TKI EGFR) will progress after 6-18 months of treatment following the emergence of resistance to treatment (Johnson *et al.,* 2005 ; Wong 2008). Several mechanisms associated with acquired resistance have been described, such as secondary EGFR mutation (T790M) or amplification of the *c-MET* in the case of EGFR TKIs (Toschi *et al.,* 2010 ; Sequist *and al.,* 2011). The management of secondary resistance therefore requires iterative evaluation of the tumor genotype.

**The aim of this project is to assess the clinical impact of circulating tumor DNA in metastatic NSCLC as early prognostic and predictive marker of response to chemotherapy (conventional or targeted) and analysis are changing over time.**

The plasma or serum may contain a small amount of free DNA, to the concentration of a few ng / mL. This free circulating DNA (ADNlc) is a double stranded DNA, fragmented whose plasma half-life is too short to about 16 min (Diehl *et al.,* 2005 ; Lecomte *et al.,* 2010). His presence is physiological but its concentration increases significantly during tumor processes and a fraction of the free DNA is then at least partially of malignancy, it is called circulating tumor DNA (ctDNA) (Lecomte *et al.,* 2010). Several studies have shown that patients with NSCLC had a much higher level of cDNA that a healthy individual (Yoon *et al.,* 2009 ; Lee *et al.,* 2011). In 1994, Sorenson demonstrates the neoplastic origin of circulating DNA in patients with pancreatic cancer, highlighting the same *KRAS* mutation in circulating DNA and in the tumor. Thus mutations, gene amplifications, chromosomal loss and translocations found in tumors can be found in the cDNA and sign the presence of ctDNA. In this case, the patient is his own control and detection of tumor molecular abnormalities in the blood confirmed the presence of ctDNA. Based on all these data, the analysis of the cDNA appears as an attractive method for the development of non-invasive tests for diagnosis, prognosis, monitoring or detection of early relapse. The ctDNA is a readily available tumor material in clinical practice since a simple venipuncture 5mL enough and can be seen regularly in the monitoring of patients.

The search for tumor alterations in plasma requires the development of robust enough molecular techniques (the amount of DNA is low) and sensitive (the ctDNA is contaminated with cDNA). Several studies have already proven that it is possible to characterize and quantify this ctDNA in several tumor types (Lecomte *et al.,* 2010 ; Schwarzenbach *et al.,* 2011).

Today the lack of sensitivity of the techniques used is the major limitation for detecting ctDNA in the plasma. Conventional methods of the security sensitivity thresholds of 0.1-1% (Didelot *et al.,* 2012). These comprehensive analysis technologies are simple to implement and compatible without technological optimization in plasma care routine analysis. They will be tested by first intention. However, the quantification of the report mutated alleles is difficult and the sensitivity may be insufficient if the ctDNA is present in very small quantities. This procedure is based on the segregation of DNA individual target molecules in millions of independent microdroplets with the order volumes of a few picoliters to reveal the presence of a molecule of interest (Pekin *and al.,* 2011 ; Zhong *et al.,* 2011). This technique has been validated by demonstrating the ability to quantitatively detect and accurately up to 0.0005% of mutated sequence in biological samples. The use of this original proceeding in this protocol should achieve a quantitative monitoring of the presence of genotyping ctDNA after the primary tumor, monitor the decrease in ctDNA during medical treatment and to detect more early relapse or treatment failure. Included patients for whom no genetic alteration is identified on *EGFR,* *KRAS,* *BRAF,* *PIK3CA,* *ALK,* and *HER2* will be analyzed on the new ION TORRENT generation sequencer to search for another biomarker using the kit Ampliseq Cancer Panel (Applied biosystems).

In total, characterization and quantification of ctDNA need to be assessed with two objectives:

- Examine its prognostic value in metastatic lung cancer

- Early identification of the molecular causes of secondary resistance.

**Primary objective**

Assess whether the detection of ctDNA before treatment initiation is a prognostic factor for overall survival after adjustment for other prognostic factors

**Secondary objectives**

Assess whether the amount of cDNA and ctDNA at treatment initiation is a prognostic factor for overall survival after adjustment for other prognostic factors

To evaluate if the decrease in cDNA and ctDNA during treatment is a prognostic factor for overall survival after adjustment with other prognostic factors

Assess whether the amount of ctDNA at best response is a prognostic factor for overall survival

Determine if the detection of *EGFR* T790M in ctDNA or amplification of cMet, before and / or under TKI EGFR is associated with resistance to treatment

**Inclusion criteria**

- Patients with histologically documented NSCLC

- IIIB Stage IV

- Beginner first-line treatment.

- Age ≥18 years

- Written informed consent

**Exclusion criteria**

- Patients unable to undergo the medical follow-up study for geographical, social or psychic

- Patient Trust

- Patient unable to read, understand and sign the information sheet and consent form

- Patient not affiliated to the social security scheme

- Previous history of cancer older than 5 years, except basal cell carcinoma skin healed or carcinoma *in situ* of the cervix cured.

**Description Search**

- Screening of tumor somatic alterations in primary or metastatic samples will be performed before starting treatment in the routine care, as recommended by the French National Cancer Institute: *KRAS*, *BRAF*, *EGFR*, *HER2*, *PI3KCA*, *ALK*. This analysis shall be made in the laboratory of biochemistry, UF Pharmacogenetics and Molecular Oncology of European Georges Pompidou Hospital (Didelot *et al.,* 2012)

- Blood sample of 3 tubes of 5 ml is performed during biological assessments of current care during consultations or chemotherapy courses.

- Circulating DNA extraction Research and absolute quantification ctDNA by earlier techniques developed and published by the laboratory (Didelot *et al, 2012;* Beijing *et al,* 2012; Didelot *et al, 2013)* The extraction, dosing and analysis of circulating tumor DNA will be realized in U775 (Pr Laurent-Puig).

**Performance of the study**

***Nature and frequency of examinations and biological samples***

At diagnosis a tumor tissue sample (biopsy or excision surgery) will be used to perform pathological examination and molecular biology. After confirming the diagnosis of NSCLC and signature of consent, the patient may be included in the study.

Quantification of cDNA and demonstration of ctDNA diagnosis and during treatment will be performed on blood test performed during usual follow-up. Three tubes of 5 ml are taken at treatment initiation, between the 3 ^rd^ and 4 ^th^ weeks between the 8 ^th^ and 9 ^th^ weeks, between 12 ^th^ and 14 ^th^ weeks and then every 10 to 12 weeks under the usual clinical monitoring.

***Clinical and radiographic examinations at baseline and during follow-up visits:***

Clinical and radiological monitoring will be performed following local guidelines, ie a thoraco-abdominopelvic CT evaluated according to RECIST 1.1 criteria before starting treatment and then every two months during treatment.

***Data collection***

- 2 letters of the name, the first letter of the first name

- Month and year of birth

- Gender

- Asian Origin of parents (yes / no) (Shigematsu H, *et al.,* 2005)

- Smoking (smoker / ex-smoker / non-smoker (<100 cigarettes / life)

- Initial performance status (WHO): 0/1/2/3/ 4

- Number of metastatic website

- Brain injury (metastasis or meningeal carcinomatosis)

- Histological type of the tumor: squamous / adenocarcinoma / large cell / other

- Data on the initial tissue sample on which analysis are made of the platform:

collection date, nature of tissue (endoscopy, surgery, transthoracic puncture, other)

Result and analysis Date:

• For EGFR, KRAS, BRAF, PI3KCA, ERBB2: transferred / type of mutation / unmutated / unknown / not done

• To EML4-ALK: translocated / non translocated / unknown / not done

- Data on the venous sample on which analysis are made of the platform

Date

Presence / Absence of cDNA; cDNA number of copies / mL

Presence / Absence of ctDNA; ctDNA number of copies / mL

The samples will be kept the time of the study and will not be used for other research purposes. They will be destroyed at the end of the study and not a collection of biological samples. The purpose of this work is to improve routine care.

- Treatments received by the patient:

adjuvant

Number of metastatic line chemotherapy

EGFR TKI: start date of treatment, end of treatment, number of cycles

- Response RECIST 1.1 after 2 months of treatment: partial response, stable disease, progression, complete response, unevaluated

- Date of death

**Statistical aspects:**

This study is a preliminary study arbitrary recruitment of 100 patients is planned, consistent with the recruitment of 2 years

- Statistical analysis: A survival model will be used to evaluate prognostic factors including the parameters usually associated with risk of recurrence and death. The associations will be tested in multivariate analysis in a Cox model, and the circulating DNA levels will be treated as a continuous variable. Survival curves progression free and overall survival at the point of time will be developed using the technique of Kaplan-Meier survival differences will be tested with the log-rank test.

**Duration of the study:**

Inclusion Duration: 2 years

Time Tracking: Up and progression than 18 months from the inclusion

**Technical and regulatory classification of research:**

This is a project to improve clinical care: the collection of additional blood is the only examination performed outside the usual care. The simple blood test is not considered as an intervention, it is therefore a non-interventional research. Besides sampling will be conducted on the venous access device used to care if this current.

**Expected results**

Technical feasibility of ctDNA monitoring in lung cancer

Identifying a prognostic marker

Interest of ctDNA for identifying alterations acquired resistance

***Primary endpoint***

Presence of ctDNA to initiation of treatment

***Secondary endpoints***

cDNA and ctDNA quantity / mL at treatment initiation

cDNA and ctDNA quantity / mL at the time of best response

Presence of molecular alterations resistance (T790M mutation, amplification of cMet) initially and / or during follow-up and overall survival

***Expected benefits for patients***

Noninvasive evaluation and early response

Noninvasive detection of secondary resistance mutations. To date if this research is useful to support a re-biopsy is needed.

**Technical aspects - Regulatory**

The research project will be conducted in accordance with the protocol, laws and regulations in force.

***1. Patient Protection Committee***

The ethic committee “Ile de France 2” issued an opinion December 2, 2013

***2. CCTIRS & CNIL***

The processing of information is conducted for the therapeutic monitoring of patients. It will be performed within the same service or services between the same care facility (Georges Pompidou European Hospital, Medical Oncology and services UF biochemistry service Pharmacogenetics and Molecular Oncology) in part data collected as part of the medical care by staff who provide such monitoring. The authorization request to the CNIL is not necessary, a normal declaration will be made. (Practical guide for health professionals (2011) Sheet No. 13).

The opinion of the Advisory Committee on Information Processing in Research in the field of Health is not necessary because there is no formation of a collection of biological samples and the study is conducted in the context of care between services of the same health facility by professionals bound by professional secrecy.

***3. Patient Information***

Patients will be informed individually about the study by their referent oncologist who hands them on this occasion a memorandum and offers them to participate in the study. After thinking if the patient agrees to participate will give its consent in writing.

**References**

Diehl F, Li M, Dressman D, et al. Detection and quantification of mutations in the plasma of patients with colorectal tumors. USA Proc Natl Acad Sci 2005; 102: 16368-16373.

Didelot A, Le Corre D, Luscan A, et al. Competitive allele specific TaqMan PCR for KRAS, BRAF and EGFR mutation detection in clinical formalin fixed paraffin embedded samples. Exp Mol Pathol 2012; 92: 275-80.

Didelot A, Kotsopoulos SK, Lupo A, et al. Multiplex-picoliter droplet digital PCR for quantitative assessment of DNA integrity in clinical samples. Clin Chem 2013; 59: 815-23.

S Guerin Hill and C. The epidemiology of cancer in France in 2010, compared with the United States. 2010 Cancer Bulletin; 97: 47-54.

Goldstraw P, Ball D, Jett JR, et al. Non-small-cell lung cancer. Lancet 2011; 378: 1727-1740.

BE Johnson and Janne P A. Epidermal growth factor receptor mutations in patients with non-small cell lung cancer. Cancer Res 2005; 65: 7525-7529.

Kwak EL, Bang YJ, Camidge DR, et al. Anaplastic lymphoma kinase inhibition in non-small-cell lung cancer. N Engl J Med 2010; **363:** 1693-703.

Lecomte T, Ceze N, E and Dorval Laurent-Puig P. Circulating free DNA tumor and colorectal cancer "Gastroenterol Clin Biol 2010; 34:. 662-681.

YJ Lee, KA Yoon, Han JY, et al. Circulating cell-free DNA in plasma of never smokers with advanced lung adenocarcinoma receiving send gefitinib or standard chemotherapy as first-line therapy. Clin Cancer Res 2011; 17: 5179-5187.

Lynch TJ, Bell DW, Sordella R, et al. Activating mutations in the epidermal growth factor receptor Underlying responsiveness of non-small-cell lung cancer to gefitinib. N Engl J Med 2004; 350: 2129-2139.

Pekin D, Skhiri Y, Baret JC, et al. Quantitative and sensitive detection of unusual mutations using droplet-based microfluidics. Lab Chip, 2011; 11: 2156-2166.

Schwarzenbach H, Hoon DS and Pantel K. Cell-free nucleic acids as biomarkers in cancer patients. Nat Rev Cancer 2011; 11: 426-437.

Sequist LV, Waltman BA, Dias-Santagata D, et al. Genotypic and histological Evolution of lung cancers acquiring resistance to EGFR inhibitors. Sci Transl Med 2011; 3: 75ra26.

Shepherd FA, Rodrigues Pereira J, Ciuleanu T, et al. "Erlotinib in Previously Treated Non-small-cell lung cancer." N Engl J Med 2005; 353: 123-132.

Shigematsu H, Lin L, Takahashi T, et al. Clinical and biological features associated with epidermal growth factor receptor gene mutations in lung cancers. J Natl Cancer Inst 2005; 97: 339-46.

Toschi L and F. Cappuzzo Impact of biomarkers is non-small cell lung cancer treatment. Target Oncol 2010; 5: 5-17.

Wong K K. Searching for a magic bullet in NSCLC: the role of epidermal growth factor receptor mutations and tyrosine kinase inhibitors. Lung Cancer 2008; 60 Suppl 2: S10-18.

Yoon, KA, S. Park, SH Lee, JH Kim and JS Lee. Comparison of circulating plasma DNA levels entre lung cancer patients and healthy controls. J Mol Diagn 2009; 11: 182-185.

Zhong Q, Bhattacharya S, Kotsopoulos S, et al. Multiplex digital PCR: breaking the one target per color barrier of quantitative PCR. Lab Chip, 2011; 11: 2167-2174.

Date: 05-08-2013

**signatures**
