# Supplementary material for: Base-Position Error Rate Analysis of Next-Generation Sequencing Applied to Circulating Tumor DNA in Non-Small Cell Lung Cancer: A Prospective Study
Source: PLoS Med. 2016 Dec 27;13(12):e1002199. doi: 10.1371/journal.pmed.1002199 (PMC5189949; doi:10.1371/journal.pmed.1002199)
Supplement: S2 Text — (DOC) [file pmed.1002199.s010.doc]

STROBE Statement—checklist of items that should be included in reports of observational studies

|  | Item No | Recommendation | Checklist filled in by the authors |
| --- | --- | --- | --- |
| **Title and abstract** | 1 | (*a*) Indicate the study’s design with a commonly used term in the title or the abstract | Title and abstract mention “a prospective study” |
|  |  | (*b*) Provide in the abstract an informative and balanced summary of what was done and what was found | In the abstract, we mentioned the number of patients and the results based on primary objective |
| Introduction | | |  |
| Background/rationale | 2 | Explain the scientific background and rationale for the investigation being reported | Done in the Introduction |
| Objectives | 3 | State specific objectives, including any prespecified hypotheses | Done in the Introduction |
| Methods | | |  |
| Study design | 4 | Present key elements of study design early in the paper | Done in Materials and Methods, subsection “Patients and tumor material” |
| Setting | 5 | Describe the setting, locations, and relevant dates, including periods of recruitment, exposure, follow-up, and data collection | Done in Materials and Methods, subsection “Patients and tumor material” and subsection “Statistical analysis” |
| Participants | 6 | (*a*) *Cohort study*—Give the eligibility criteria, and the sources and methods of selection of participants. Describe methods of follow-up  *Case-control study*—Give the eligibility criteria, and the sources and methods of case ascertainment and control selection. Give the rationale for the choice of cases and controls  *Cross-sectional study*—Give the eligibility criteria, and the sources and methods of selection of participants | Considering a cohort study, sources of participants, eligibility criteria and non inclusion criteria are described in Materials and Methods, subsection “Patients and tumor material” |
|  |  | (*b*) *Cohort study*—For matched studies, give matching criteria and number of exposed and unexposed  *Case-control study*—For matched studies, give matching criteria and the number of controls per case | NA |
| Variables | 7 | Clearly define all outcomes, exposures, predictors, potential confounders, and effect modifiers. Give diagnostic criteria, if applicable | The main outcome is overall survival, and is described in Materials and Methods, subsection “Statistical analysis”. Confounding prognostic makers are listed in Materials and Methods, subsection “Patients and tumor material”. The main effect modifier is a positive ctDNA at baseline, as defined Materials and Methods, subsection “Statistical analysis” and described in the Results and Fig 2. |
| Data sources/ measurement | 8* | For each variable of interest, give sources of data and details of methods of assessment (measurement). Describe comparability of assessment methods if there is more than one group | Measurements of ctDNA has been performed by two independent technics, which are described in Materials and Methods, subsections “Digital PCR: emulsion generation, thermal-cycling, and droplet analyses” and “Next-generation sequencing analyses and protocol”. Between technics agreement is discussed in the Results and in Fig 2. |
| Bias | 9 | Describe any efforts to address potential sources of bias | Biases are discussed in Materials and Methods, subsection “Patients and tumor material”. |
| Study size | 10 | Explain how the study size was arrived at | Sample size calculation is described in Materials and Methods, subsection “Statistical analysis”. |
| Quantitative variables | 11 | Explain how quantitative variables were handled in the analyses. If applicable, describe which groupings were chosen and why | To study the impact of absolute ctDNA quantity, patients were categorized into tertiles (n=35 patients in each tertile) defining low (<0.027 ng/mL), intermediate (0.027-0.50 ng/mL) and high (>0.50 ng/mL) level groups (Materials and Methods, subsection “ctDNA absolute quantification”).  Tumor burden was estimated using the RECIST baseline sum of longest diameters and categorized as low ≤ 7.5 cm or high > 7.5 cm as previously described in published study (Materials and Methods, subsection “Patients and tumor material”) |
| Statistical methods | 12 | (*a*) Describe all statistical methods, including those used to control for confounding | Baseline ctDNA was considered as positive if detected by NGS or by dPCR. The statistical analyses were performed on the per-protocol population composed of all patients with at least one molecular alteration identified in the tumor or the initial plasma sample by NGS.  The Cox proportional-hazards regression model was used to perform univariate and multivariate analyses with 95% confidence interval (CI). Multivariate analysis was performed using variables associated with the outcome in univariate analysis at a P value of < .05. |
|  |  | (*b*) Describe any methods used to examine subgroups and interactions | Subgroups were compared as followed: survival curves were compared with log-rank test, proportions were compared with Fisher’s exact test and continuous variables using Mann-Whitney test.  We did not perform interaction tests. |
|  |  | (*c*) Explain how missing data were addressed | Missing ctDNA evaluations are detailed page 8: Among 109 patients eligible for ctDNA follow-up, 85 underwent their first evaluation at 6±2 weeks, 13 received no follow-up evaluation, 7 had their first evaluation either before one month or after 2 months, and 4 died within the first month.  Patients with no evaluation were excluded from survival analysis. |
|  |  | (*d*) *Cohort study*—If applicable, explain how loss to follow-up was addressed  *Case-control study*—If applicable, explain how matching of cases and controls was addressed  *Cross-sectional study*—If applicable, describe analytical methods taking account of sampling strategy | Follow-up of patients was performed until death. Patients were censured at last-follow up or at the cut-off date for analysis May 2016. After a median follow-up of 18.8 months, 94 and 63 events occurred for progression-free survival (PFS) and OS, respectively. |
|  |  | (*e*) Describe any sensitivity analyses | The analytical sensitivity of the method has been previously validated and described in Pécuchet N, Rozenholc Y, Zonta E, Pietraz D, Didelot A, Combe P, et al. Analysis of Base-Position Error Rate of Next-Generation Sequencing to Detect Tumor Mutations in Circulating DNA. Clinical Chemistry. Clinical Chemistry; 2016; clinchem.2016.258236. doi:10.1373/clinchem.2016.258236 |

Continued on next page

| Results | | | Checklist |
| --- | --- | --- | --- |
| Participants | 13* | (a) Report numbers of individuals at each stage of study—eg numbers potentially eligible, examined for eligibility, confirmed eligible, included in the study, completing follow-up, and analysed | The number of patients are given for each stage of the study in Figure 1 and Table 2. |
|  |  | (b) Give reasons for non-participation at each stage | Done in Figure 1. |
|  |  | (c) Consider use of a flow diagram | Done in Figure 1. |
| Descriptive data | 14* | (a) Give characteristics of study participants (eg demographic, clinical, social) and information on exposures and potential confounders | Table 1 |
|  |  | (b) Indicate number of participants with missing data for each variable of interest | Table 2 |
|  |  | (c) *Cohort study*—Summarise follow-up time (eg, average and total amount) |  |
| Outcome data | 15* | *Cohort study*—Report numbers of outcome events or summary measures over time | After a median follow-up of 18.8 months, 94 and 63 events occurred for progression-free survival (PFS) and OS, respectively. |
|  |  | *Case-control study—*Report numbers in each exposure category, or summary measures of exposure |  |
|  |  | *Cross-sectional study—*Report numbers of outcome events or summary measures |  |
| Main results | 16 | (*a*) Give unadjusted estimates and, if applicable, confounder-adjusted estimates and their precision (eg, 95% confidence interval). Make clear which confounders were adjusted for and why they were included | Table 3.  The Cox proportional-hazards regression model was used to perform univariate and multivariate analyses with 95% confidence interval (CI). Multivariate analysis was performed using variables associated with the outcome in univariate analysis at a P value of < .05. |
|  |  | (*b*) Report category boundaries when continuous variables were categorized | To study the impact of absolute ctDNA quantity, patients were categorized into tertiles (n=35 patients in each tertile) defining low (<0.027 ng/mL), intermediate (0.027-0.50 ng/mL) and high (>0.50 ng/mL) level groups.  Tumor burden was estimated using the RECIST baseline sum of longest diameters and categorized as low ≤ 7.5 cm or high > 7.5 cm as previously described in published study. |
|  |  | (*c*) If relevant, consider translating estimates of relative risk into absolute risk for a meaningful time period |  |
| Other analyses | 17 | Report other analyses done—eg analyses of subgroups and interactions, and sensitivity analyses |  |
| Discussion | | |  |
| Key results | 18 | Summarise key results with reference to study objectives | Done in Discussion |
| Limitations | 19 | Discuss limitations of the study, taking into account sources of potential bias or imprecision. Discuss both direction and magnitude of any potential bias | Done in Discussion |
| Interpretation | 20 | Give a cautious overall interpretation of results considering objectives, limitations, multiplicity of analyses, results from similar studies, and other relevant evidence | Done in Discussion |
| Generalisability | 21 | Discuss the generalisability (external validity) of the study results | Done in Discussion |
| Other information | | |  |
| Funding | 22 | Give the source of funding and the role of the funders for the present study and, if applicable, for the original study on which the present article is based | Done |

*Give information separately for cases and controls in case-control studies and, if applicable, for exposed and unexposed groups in cohort and cross-sectional studies.

**Note:** An Explanation and Elaboration article discusses each checklist item and gives methodological background and published examples of transparent reporting. The STROBE checklist is best used in conjunction with this article (freely available on the Web sites of PLoS Medicine at http://www.plosmedicine.org/, Annals of Internal Medicine at http://www.annals.org/, and Epidemiology at http://www.epidem.com/). Information on the STROBE Initiative is available at www.strobe-statement.org.
